# Combining genetic risk score with artificial neural network to predict the efficacy of folic acid therapy to hyperhomocysteinemia

**DOI:** 10.1038/s41598-021-00938-8

**Published:** 2021-11-02

**Authors:** Xiaorui Chen, Xiaowen Huang, Diao Jie, Caifang Zheng, Xiliang Wang, Bowen Zhang, Weihao Shao, Gaili Wang, Weidong Zhang

**Affiliations:** 1 Department of Epidemiology, School of Public Health, Zhengzhou University, Zhengzhou, 450001 Henan China; 2 The University of Glasgow, Glasgow, G12 8QQ Scotland

**Keywords:** Computational biology and bioinformatics, Genetics, Biomarkers, Health care, Medical research, Molecular medicine, Risk factors

## Abstract

Artificial neural network (ANN) is the main tool to dig data and was inspired by the human brain and nervous system. Several studies clarified its application in medicine. However, none has applied ANN to predict the efficacy of folic acid treatment to Hyperhomocysteinemia (HHcy). The efficacy has been proved to associate with both genetic and environmental factors while previous studies just focused on the latter one. The explained variance genetic risk score (EV-GRS) had better power and could represent the effect of genetic architectures. Our aim was to add EV-GRS into environmental factors to establish ANN to predict the efficacy of folic acid therapy to HHcy. We performed the prospective cohort research enrolling 638 HHcy patients. The multilayer perception algorithm was applied to construct ANN. To evaluate the effect of ANN, we also established logistic regression (LR) model to compare with ANN. According to our results, EV-GRS was statistically associated with the efficacy no matter analyzed as a continuous variable (OR = 3.301, 95%CI 1.954–5.576, *P* < 0.001) or category variable (OR = 3.870, 95%CI 2.092–7.159, *P* < 0.001). In our ANN model, the accuracy was 84.78%, the Youden’s index was 0.7073 and the AUC was 0.938. These indexes above indicated higher power. When compared with LR, the AUC, accuracy, and Youden’s index of the ANN model (84.78%, 0.938, 0.7073) were all slightly higher than the LR model (83.33% 0.910, 0.6687). Therefore, clinical application of the ANN model may be able to better predict the folic acid efficacy to HHcy than the traditional LR model. When testing two models in the validation set, we got the same conclusion. This study appears to be the first one to establish the ANN model which added EV-GRS into environmental factors to predict the efficacy of folic acid to HHcy. This model would be able to offer clinicians a new method to make decisions and individual therapeutic plans.

## Introduction

The process of data digging is defined as using techniques to discover hidden patterns and correlations from complex datasets^1^. And it is described as the method to construct predictive models based on relationships in large datasets and the discovery of underlying patterns.

Artificial neural network (ANN) is one of the main tools to dig data. And it has a complex computational structure that is inspired by the human brain and nervous system^2^. The structure of ANN consists of the input layer, output layer, and hidden layer. Three layers compose the units which transform the information of the input layer into something that we expect to get in the output layer^3^. ANN is an exceptional tool to identify the correlations from complex and numerous datasets to extract meaningful information and recognize relationships^4–6^. Therefore, ANN can be used to incorporate intricate associations among numerous variables into algorithms. In the medical fields, recent researches concerning ANN have constructed numbers of prediction models, such as survival prediction for gastric cancer^4^, the length of staying in an intensive care unit (ICU)^5^, and the risk prediction of congenital heart disease among pregnant women^7^. However, none of them have employed ANN to investigate the association between folic acid and hyperhomocysteinemia (HHcy).

Homocysteine (Hcy) is a nonessential amino acid that is produced by methionine’s metabolism to cysteine^8,9^. As for HHcy, we defined it as the fasting plasma total homocysteine (tHcy) ≥ 15 μmol/L^10–12^. Preliminary studies have confirmed HHcy is significantly related to cardiovascular disease and maybe an independent important risk factor for CVD, Alzheimer’s disease, neural tube defects, inflammatory bowel disease, and several non-communicable diseases^13–15^. And oral folic acid is one of the most common interventions in HHcy treatment to reduce Hcy’s concentration^16^. While after 90 days’ supplementation of oral 5-mg folic acid in our preliminary studies, over 40% HHcy participants failed to reduce to the normal level. Thus, an ANN model to predict the efficacy of folic acid therapy to HHcy is very necessary and useful for clinical practice^17,18^.

According to several previous studies, genetic architectures and clinic biochemical indexes both have an important role in the efficacy while most studies just focused on the latter one^19^. Therefore, we’d like to add genetic into environmental factors to establish an ANN model. Previous studies had revealed a number of signal nucleotide polymorphisms (SNPs) associated with the folic acid’ efficacy of HHcy^20^. In addition, several studies demonstrated that explained variance genetic risk score (EV-GRS) which considered the effect of single nucleotide polymorphisms (SNPs) and minor allele frequency (MAF) comprehensively could be applied to explore the relationships between genetic architectures and complex diseases^21^. And the article also proved that EV-GRS has higher accuracy and better power. Therefore, we calculated the EV-GRS to represent genetic risk factors and added EV-GRS into environmental factors to establish an ANN model to construct a prediction model to predict the efficacy of folic acid therapy to HHcy.

Although there are many algorithms to construct ANN, we undertook the multilayer perception (MLP) which is one of the most typical supervised studying algorithms in which a very small number of parameters can predict outcomes^22^. What’s more, MLP can be used in packaging software including SPSS (IBM Corporation, New York, USA) and JMP (SAS Institute, Cary, UC, USA). Because it doesn’t need complex programming, this methodology is expected to be very easily adaptable by clinicians and pharmacists. Though MLP isn’t new, the approach to apply it to the efficacy prediction of folic acid to HHcy is novel^23,24^.

The objective of our present study was to add EV-GRS into environmental factors to construct an ANN prediction model to predict the efficacy of folic acid therapy to HHcy. Meanwhile, we constructed the traditional logistic regression (LR) model and compared the effects of the ANN and LR model by the area under the receiver operating characteristic curve (AUC), accuracy, precision, sensitivity, and specificity. Then we could construct a more accurate model to provide a more reasonable individualized treatment plan for HHcy patients.

## Materials and methods

### Study design and participants

We conducted a prospective study and evolved 1033 HHcy patients (tHcy ≥ 15 μmol/L) who had measured the plasma Hcy level in the Department of Neurology in the Fifth Affiliated Hospital of Zhengzhou University from July to December 2014. Our preliminary study explained that there was a significant difference in folic acid’s effective rate among 638 HHcy patients (175 subjects were excluded as they lost to follow-up and poor compliance) who had good or moderate compliance.

Then we randomly divided the 638 patients into a development set (n = 444, 70%) and a validation set (n = 194, 30%), and the development set was used to construct ANN predictive model and the validation set was separated for evaluation of the final model.

The research was approved by the Ethics Review Committee of the Life Science of Zhengzhou University. All of the subjects or relatives signed informed consent.

### SNPs selection and genotyping

The 638 patients who had good compliance were extracted genomic DNA following the instructions of whole blood genomic DNA extraction kit (Bio Teke, Beijing, China). We got the SNPs’ information from the HapMap database (from http://hapmap.ncbi.nlm.nih.gov/). And we screened the tag SNPs with Haplo View 4.2 software (from https://www.broadinstitute.org/haploview). Our inclusion criteria were as follows: (1) check markers, minor allele frequency (MAF) > 0.05 and rescore markers; (2) Tagger, r^2^ > 0.8 and run Tagger; and (3) get the functional SNP or SNP which induced changes in protein activity. Then we used Sequenom's MassArray system (San Diego, CA, USA) to detect the genotypes and alleles.

In our study, we tested 23 previously studied SNPs that may affect the efficacy of oral folic acid therapy. And the detailed information was presented in Supplementary Table S1. The SNPs all had MAF > 0.05 and did not deviate from the Hardy–Weinberg equilibrium (HWE). Then based on the candidate SNPs, we conducted a multivariate logistic regression to screen out the SNPs that were significantly different between the success group and failure group. Finally, we enrolled 6 SNPs (MTHFR rs1801133, MTHFR rs1801131, MTHFD rs2236225, MTRR rs1801394, CBS rs706209, BHMT rs3733890) to calculate EV-GRS.

### Explained variance-genetic risk score

The EV-GRS was a method that considered both the effects of Minor Allele Frequency (MAF) and SNP^21^. According to the definition, EV-GRS thought SNP and MAF both have a very important impact on the outcome in each SNP locus^25^. The calculation formula and the model are as follows:

$$\begin{aligned} &\upomega _{i} = \ln \left( {OR_{i} } \right)\sqrt {2MAF_{I} \left( {1 - MAF_{I} } \right)} \\ & {\text{GRS}} = \mathop \sum \limits_{i = 1}^{n} \omega_{i} G_{i} \\ \end{aligned}$$

where n was the number of SNPs, ln (OR_i)_ was the weight of the ith SNP locus, MAF_i_ was the MAF of the ith SNP locus, G_i_ was the ith risk allele of SNP locus.

### Optimal independent variables selection and the LR model establishment

The LR and ANN models were developed based on the identification of independent predictors for the efficacy of folic acid to HHcy. Determination of the independent risk factors was achieved through LR analysis. Firstly, we undertook binary LR analysis in development set to determine meaningful factors. Then based on the results of binary LR analysis, we used multivariable LR analysis to screen out the statistically meaningful risk factors as the independent variables of LR and ANN models in development set.

For the LR model, its construction was, to sum up, relevant risk factors which were also multiplied by their weights to predict the efficacy of oral folic acid to HHcy patients. We constructed the LR model in both development set and validation set.

### Establishment of ANN model

For the establishment of the ANN model, we used the 3-layer, feed-backward neural network which includes the input nodes, a hidden layer, and the output nodes. As for the MLP, it consists of an input layer containing risk factors’ information and followed by the hidden layer which interacts with the variables that are eventually transferred to the output layer. The neuron nodes’ number in the input layer depends on the number of evolving independent variables, whereas neuron nodes’ number of the output layer is associated with the number of outcomes that need to predict^23,26^. The number of neuron nodes in the hidden layer ranged from 1 to 50.

We set the training’s type as a batch, the optimal algorithm as scaled conjugate gradient, the initial Lambda as 0.0000005, the initial Sigma as 0.00005, the interval center as 0, and the interval offset as 0.5. And hyperbolic tangent function was used to activate in the hidden layer. In addition, to output the efficacy of folic acid treatment to HHcy, we used the softmax function as the activation function in the output layer. The ANN training would stop when maximum steps without any decrease in error were 1. As for other options, we used default options^27^.

The MLP’s steps are summarized as follows^23,26^: (1) information is provided to the input layer; (2) the input layer calculates a predicted output layer that is subtracted from the actual output, meanwhile, an error value is estimated; (3) then a backpropagation adjusts weights between output layer and hidden layer that works backward through a network; (4) After a backpropagation finished, the process would start again; and (5) this process would repeat until the error is minimized. The ANN model was established via the use of the SPSS Neural Network module, version 21.0 (IBM, Armonk, NY).

### Statistical analysis

We compared the baseline demographics on the development set and validation set. The continuous variables were showed as means with standard deviation and were compared by Student’s *t* test. The categorical variables were showed as the frequency with percentage and were compared by χ^2^ test. We firstly conducted the binary logistic analysis to screen out the meaningful independent variables between success and failure groups. Then based on the result of binary logistic analysis, a multinomial logistic analysis was performed to choose final meaningful variables to develop LR and ANN model.

To evaluate the predictive performance of the LR and ANN model, we plotted the receiver-operating characteristic (ROC) curve and also calculated the area under the receiver operating curve (AUC)^28^. Meanwhile, we calculated several other metrics as sensitivity, specificity, Youden’s index, and accuracy^29–31^.

All statistical analyses were performed using SPSS 21.0 (IBM Corporation, New York, USA) and MedCalc 15.2.2 (MedCalc Software, Ostend, Belgium). Two‑sided *P* < 0.05 was considered statistically significant.

## Results

### Demographic characteristics of development set and validation set

All of the 638 eligible patients with complete information were enrolled in our study. The patients were randomly divided into development set (n = 444, 70%) and validation set (n = 194, 30%). The information of demographic characteristics in two sets was shown in Table 1. As shown in Table 1, there was no statistically meaningful difference between the development set and validation set on baseline characteristics and clinical biochemical indexes.

**Table 1:** Demographic characteristics of development set and validation set.

Variables	Development set	Validation set	Sum up	χ^2^/t	P
	(n = 447)	(n = 191)	(n = 638)		
Age,(years, ${\overline{\text{X}}}$ ± S)	65.05 ± 14.88	66.22 ± 14.20	65.38 ± 14.69	1.08^a^	0.28
*Sex, n(%)*		0.098	0.755		
Male	282 (63.09)	118 (61.78)	402 (63.01)		
Female	165 (36.91)	73 (38.22)	236 (36.99)		
BMI, (kg/m^2^)	23.99 ± 2.05	23.79 ± 2.13	23.93 ± 2.07	− 1.183^a^	0.237
Smoking, n (%)	152 (34.00)	69 (36.13)	217 (34.01)	0.266	0.606
Drinking, n (%)	63 (14.09)	31 (16.23)	96 (15.05)	0.486	0.486
History, n (%)	143 (31.99)	61 (31.94)	204 (31.97)	0	0.989
Diabetics, n (%)	112(25.06)	52 (27.23)	160(25.08)	0.33	0.566
Hypertension, n (%)	241 (53.91)	111 (58.12)	351 (55.02)	0.955	0.329
Hyperlipidemia, n (%)	9 (2.01)	4 (2.09)	13 (2.04)	0.004	0.947
Stroke, n (%)	143 (31.99)	55 (28.80)	198 (31.03)	0.638	0.424
CHD, n (%)	107 (23.94)	55 (28.80)	166 (26.02)	1.667	0.197
FPG, (mmol/L, ${\overline{\text{X}}}$ ± S)	5.48 ± 5.11	5.64 ± 2.14	5.52 ± 2.08	1.363^a^	0.173
TG, (mmol/L, ${\overline{\text{X}}}$ ± S)	1.63 ± 1.13	1.53 ± 1.07	1.58 ± 1.12	1.374^a^	0.17
TC, (mmol/L, ${\overline{\text{X}}}$ ± S)	4.34 ± 1.10	4.35 ± 0.89	4.34 ± 1.01	− 0.649^a^	0.516
LDL-C, (mmol/L, ${\overline{\text{X}}}$ ± S)	2.58 ± 0.80	2.51 ± 0.72	2.55 ± 0.75	− 1.027^a^	0.305
HDL-C, (mmol/L, ${\overline{\text{X}}}$ ± S)	1.10 ± 0.33	1.13 ± 0.28	1.12 ± 0.29	0.298^a^	0.766
Hcy, (μmol/L, ${\overline{\text{X}}}$ ± S)	22.25 ± 8.77	22.17 ± 7.59	22.18 ± 8.43	-0.040^a^	0.968

*BMI* body mass index, *CHD* coronary heart disease, *FPG* fasting plasma glucose, *TG* triglycerides, *TC* total cholesterol, *LDL-C* low density lipoprotein cholesterol, *HDL-C* high density lipoprotein cholesterol, *Hcy* homocysteine.

^a^Student’s *t* test.

### The association between EV-GRS and the efficacy of folic acid treatment to HHcy

According to the algorithm of EV-GRS, we calculated the score and evaluated the relationship between EV-GRS and the efficacy of oral folic acid (Table 2). When EV-GRS was modeled as continuous variables, the association was statistically meaningful (OR = 3.301, 95%CI 1.954–5.576, *P* < 0.001).

**Table 2:** Association between EV–GRS and the efficacy of folic acid therapy to HHcy.

EV–GRS	Success group n (%)	Failure group n (%)	Crude *OR* (95% CI)	*P*	Adjusted *OR* (95% CI)^a^	*P*^a^
Continuous			2.478 (1.728–3.553)	< 0.001	3.301 (1.954–5.576)	< 0.001
**Category**						
1 (< P25)	58 (25.55)	64 (29.09)	Reference		Reference	
2 (P25-P50)	65 (28.63)	49 (22.27)	2.361 (1.293–4.310)	0.005	6.71 (2.653–16.973)	< 0.001
3 (P50-P75)	57 (25.11)	56 (25.45)	3.307 (1.806–6.508)	< 0.001	6.264 (2.450–16.013)	< 0.001
4 (≥ P75)	47 (20.70)	51 (23.18)	3.870 (2.092–7.159)	< 0.001	11.153 (4.263–29.184)	< 0.001

*OR* odds ratio.

^a^Adjusted for history, hypertension, stroke, CHD and Hcy.

Then we modeled EV-GRS as category variables to analyze the relationship. We modeled it as category variables by quartiles. Then we found that the more risk alleles participants carried, the bigger OR and the higher risk they would have to fail the treatment with or without adjustment for history, hypertension, stroke, CHD, and Hcy. When compared to the reference group (< P25), the risk of the fourth group (≥ P75) failing the treatment was significantly increased (OR = 3.870, 95%CI 2.092–7.159, *P* < 0.001). After the adjustment of history, hypertension, stroke, CHD, and Hcy, the risk was also significantly increased (OR = 11.153, 95%CI 4.263–29.184, *P* < 0.001). The results showed that EV-GRS had an intense connection with efficacy. We can recruit EV-GRS representing genetic risk factors and combine them with traditional clinical risk factors to construct the ANN prediction model.

### Screening of independent variables by logistic regression analysis

First of all, we performed binary and multivariable logistic analysis successively. The results of the binary logistic analysis showed that it was significantly different in sex, BMI, history, diabetics, hypertension, hyperlipidemia, stroke, CHD, TC, LDL-C, HDL-C, and Hcy. Then based on the results of binary logistic analysis, we enrolled the meaningful factors as independent variables and the efficacy as a dependent variable. As showing in Table 3, BMI, history, hypertension, hyperlipidemia, stroke, CHD, HDL-C, Hcy, and EV-GRS were still significantly different between the success and failure group, which would be used to establish the LR and ANN models.

**Table 3:** The multinomial logistic analysis between success group and failure group in training set.

Variables	β	OR (95%CI)	*P*
BMI	0.147	1.159 (1.003–1.339)	0.046
History, (yes vs. no)	2.308	10.050 (5.275–19.145)	< 0.001
Hypertension, (yes vs. no)	0.59	1.805 (1.015–3.210)	0.044
Hyperlipidemia, (yes vs. no)	3.085	21.858 (23.107–226.800)	0.01
Stroke, (yes vs. no)	3.303	27.186 (12.943–57.106)	< 0.001
CHD, (yes vs. no)	1.594	4.923 (2.500–9.694)	< 0.001
HDL-C, (mmol/L)	− 1.15	0.317 (0.104–0.961)	0.042
Hcy, (μmol/L)	0.084	1.088 (1.047–1.129)	< 0.001
EV-GRS	1.508	4.518 (2.277–8.964)	< 0.001

*CHD* coronary heart disease, *HDL-C* high density lipoprotein cholesterol, *Hcy* homocysteine.

### The establishment of the ANN model

**Figure 1:**
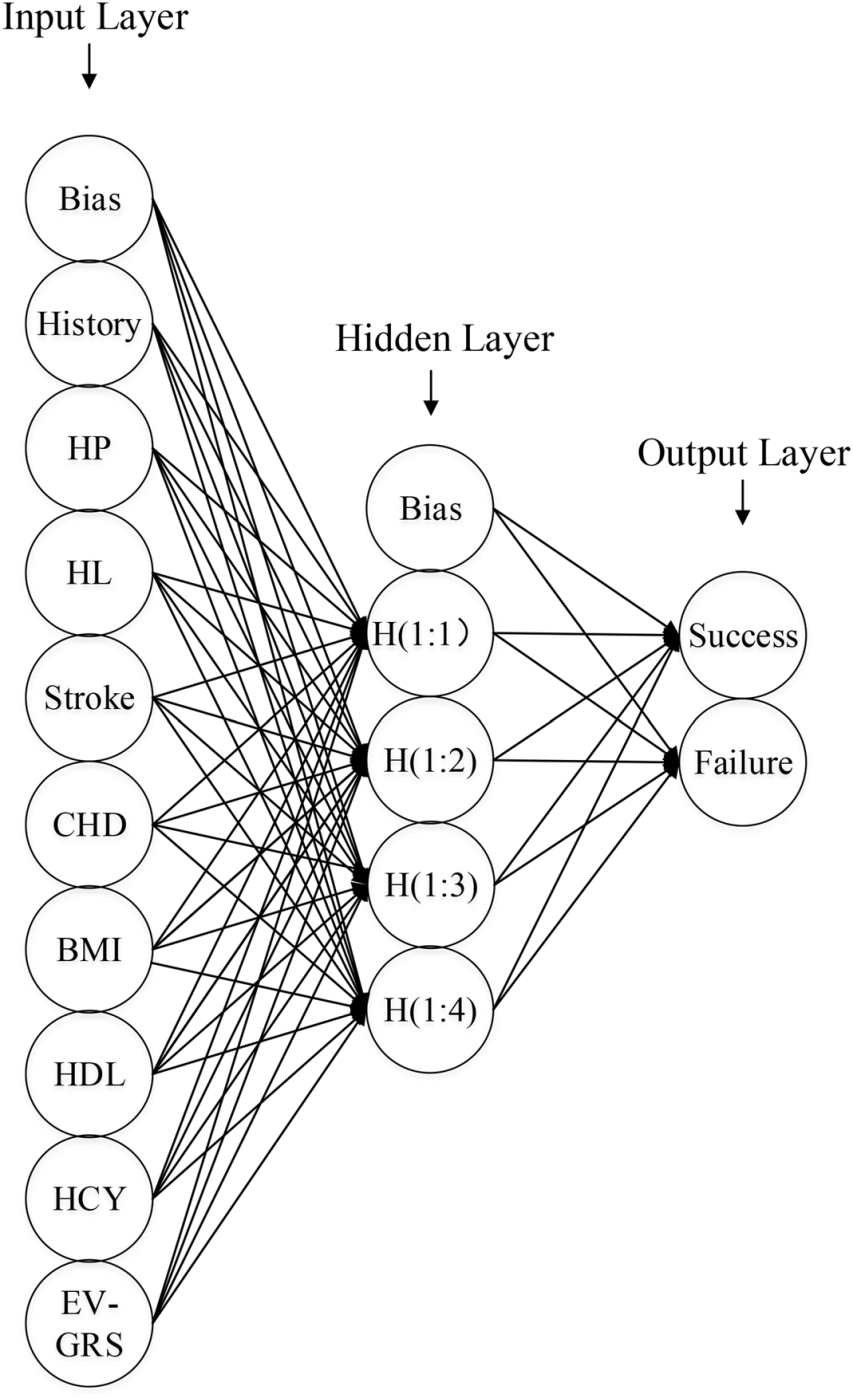
Schematic representation of the ANN model developed to predict the efficacy of folic acid therapy to HHcy.

The ANN model predicting the efficacy of folic acid to HHcy is shown in Fig. 1. Based on the multivariable logistic analysis, the nine independent variables were enrolled, and the dependent variable was the success or failure group. Our ANN model is made up of an input layer, the hidden layer, and the output layer. The input, hidden and output layers contained nine, four, and one neuron, respectively.

**Figure 2:**
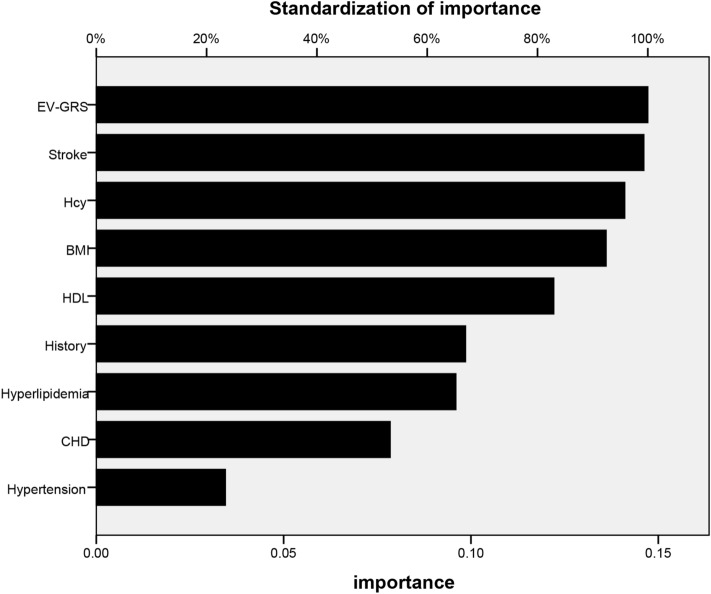
Relative importance of the 9 risk factors to the ANN model. *Hcy* homocysteine, *HDL-C* high density lipoprotein cholesterol, *EV-GRS* explained variance genetic risk score, *HL* hyperlipidemia, *CHD* coronary heart disease, *HP* Hypertension.

And the relative importance of nine independent variables in our ANN model is showed in Fig. 2 and Table 4. The top three risk factors were EV-GRS, stroke, and baseline Hcy.

**Table 4:** The importance of variables in ANN model.

Variables	Importance	Standard importance (%)	Rank
EV-GRS	0.169	100.0	1
Stroke, (yes vs. no)	0.147	87.2	2
Hcy, (μmol/L)	0.143	84.7	3
BMI	0.125	74.2	4
HDL-C, (mmol/L)	0.121	71.9	5
History, (yes vs. no)	0.119	70.6	6
Hyperlipidemia, (yes vs. no)	0.085	50.2	7
CHD, (yes vs. no)	0.074	44.1	8
Hypertension, (yes vs. no)	0.015	9.1	9

*EV-GRS* explained variance genetic risk score, *Hcy* homocysteine, *HDL-C* high density lipoprotein cholesterol, *CHD* coronary heart disease.

### The predictive capability analysis of LR and ANN model

**Figure 3:**
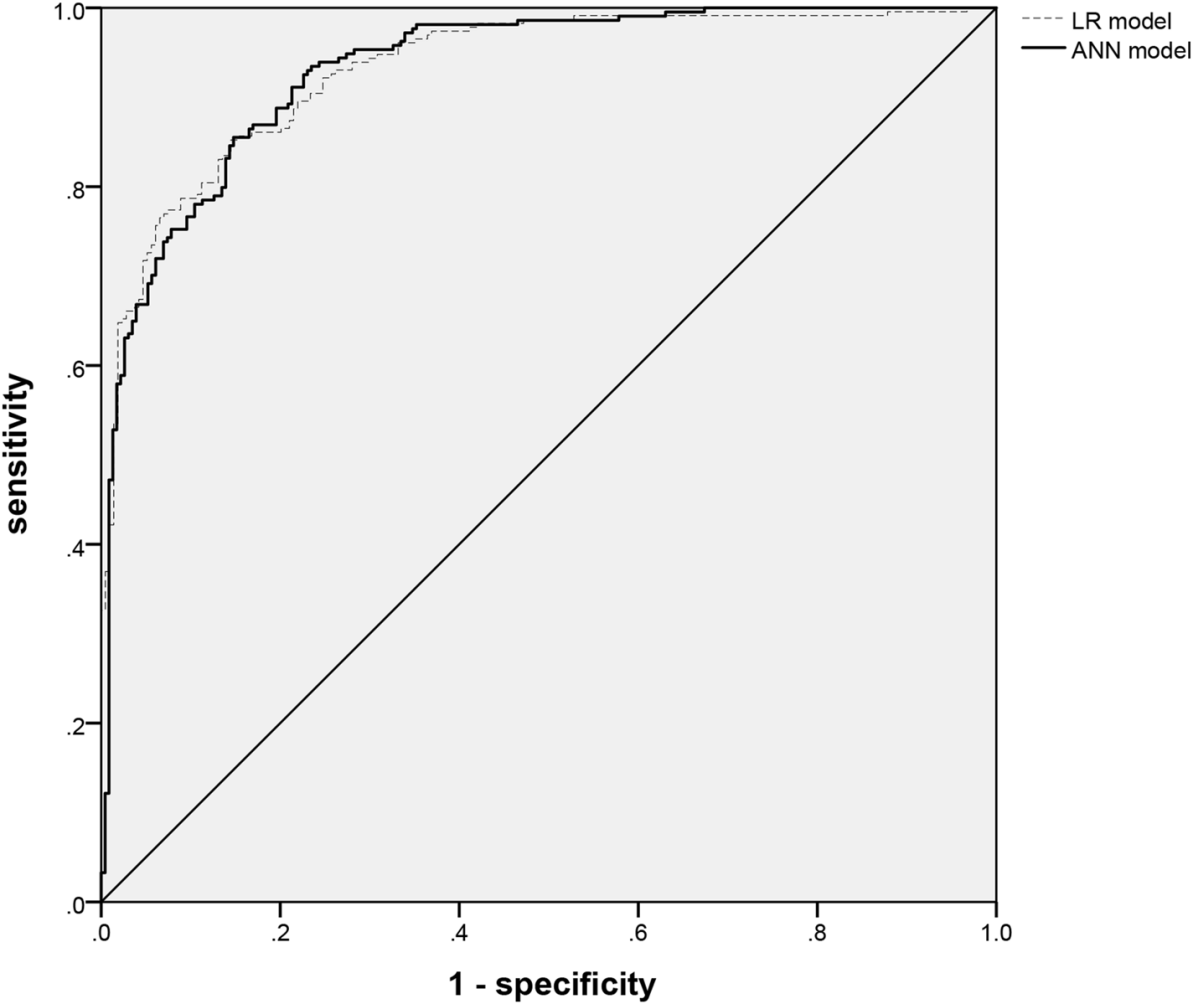
ROC curves for the ANN model to predict the efficacy of folic acid therapy to HHcy in the development set.

As presented in Fig. 3, the AUCs of the LR and ANN model were 0.910 and 0.938, individually. Both of them were above 0.9, which means that their predictive capabilities were excellent. The predictive accuracy of the ANN model was 84.78% and that of the LR model was 83.33% (Table 5). In addition, the sensitivity and specificity of our ANN model in the development set were 85.22% and 85.51%. And the sensitivity and specificity of the LR model in the development set were 86.96% and 79.91%. As presented in Table 5, the AUC, Youden’s index, and accuracy of the ANN model were all better than that in the LR model.

**Table 5:** The evaluation indicators of different predictive models in development set.

	AUC (95% *CI*)	Sensitivity(%) (95% CI)	Specificity (%) (95% CI)	Youden’s index (95% CI)	Accuracy (%) (95% CI)
Logistic regression model^a^	0.910 (0.883–0.937)	86.96 (79.06–91.33)	79.91 (74.48–83.97)	0.6687 (0.6293–0.6915)	83.33 (78.86–89.17)
ANN model^b^	0.938 (0.905–0.964)	85.22 (79.84–89.67)	85.51 (79.19–90.45)	0.7073 (0.6634–0.7527)	84.78 (79.42–90.82)

*AUC* area under the curve, *ANN* artificial neural network.

^a^When compared with Logistic regression model, there was statistical difference in AUC (*P* < 0.05).

^b^When compared with ANN model, there was statistical difference in AUC (*P* < 0.05).

Then we validated the two models in the validation set. As presented in Fig. 3, the AUCs of LR and ANN model were 0.878 and 0.900, individually. The predictive accuracy of the ANN model was 80.41% and that of the LR model was 81.96% (Table 6). In addition, the sensitivity and specificity of our ANN model were 83.16% and 80.81%. And the sensitivity and specificity of the LR model were 76.84% and 83.84%. As presented in Table 6, the AUC, Youden’s index, and accuracy of the ANN model were all better than that in the LR model, which was the same as the results in the development set.

**Table 6:** The evaluation indicators of different predictive models in validation set.

	AUC (95% CI)	Sensitivity(%) (95% CI)	Specificity (%) (95% CI)	Youden’s index (95% CI)	Accuracy (%) (95% CI)
Logistic regression model^a^	0.878 (0.830–0.925)	76.84 (71.63–81.45)	83.84 (78.32–88.50)	0.6068 (0.5734–0.6358)	80.41 (77.01–83.29)
ANN model^b^	0.90 (0.849–0.938)	83.16 (79.63–87.09)	80.81 (76.57–85.29)	0.6397 (0.6051–0.6602)	81.96 (77.24–85.02)

*AUC* area under the curve, *ANN* artificial neural network.

^a^When compared with Logistic regression model, there was statistical difference in AUC (*P* < 0.05).

^b^When compared with ANN model, there was statistical difference in AUC (*P* < 0.05).

## Discussion

To the best of our information, this is the first research to establish and validate the use of ANN which added EV-GRS into traditional clinic factors applied to the folic acid’s efficacy prediction to HHcy^5,8,11,21,29^. The EV-GRS was tested to be statistically associated with the efficacy no matter analyzed as a continuous variable (OR = 3.301, 95%CI 1.954–5.576, *P* < 0.001) or category variable (OR = 3.870, 95%CI 2.092–7.159, *P* < 0.001).In our ANN model, the accuracy was 84.78%, the Youden’s index was 0.7073 and the AUC was 0.938. The indexes above were used in several previous studies which regarded the indexes as very important performance scores as well. The AUC of our ANN model (0.938) indicated better accuracy according to the criteria reported by Akobeng. In addition, when compared with the multivariable logistic regression (LR) model, the accuracy of our ANN model (84.78%) was slightly higher than the accuracy of the multivariable LR model (83.33%). The comparison of predictive performances of ANN and LR models has been studied in several previous types of research^26,29–31^. According to a systematic review, ANN had high accuracy and was statistically different (odds ratio: 1.09)^32,33^. In other previous studies, they obtained similar conclusions^23,29–31^. Therefore, clinical application of the ANN model may be able to better predict the folic acid efficacy to HHcy than the multivariable LR model. In addition, this method can also be applied to other conditions and developed further. Meanwhile, the LR model will be appropriate if the primary aim is to extract dependent risk factors affecting folic acid efficacy to HHcy as ANN can’t screen out individual risk factors automatically^34^.

As shown in Fig. 2, the EV-GRS was extracted as the most important risk factor of efficacy prediction in ANN. EV-GRS is a popular method to explore genetic risk architectures and the relationships of many complex diseases^21^. Previous studies had revealed several signal nucleotide polymorphisms (SNPs) associated with the folic acid treatment of HHcy^17,18^. To combine numbers of SNPs’ effect, we’d like to undertake the genetic risk score (GRS) method. There are four common kinds of GRS, (1) simple count genetic risk score (SC-GRS), (2) direct logistic regression genetic risk score (DL-GRS), (3) polygenic genetic risk score (PG-GRS), and (4) explained variance weighted genetic risk score (EV-GRS)^21,35–37^. The SC–GRS just calculated the number of risk alleles across every SNP at the chosen loci. Its outcome was 0, 1, 2, 3, 4, 5, and 6. The DL-GRS and PG-GRS considered the influence of different SNPs. The EV-GRS considered both the influence of SNP and the Minor Allele Frequency (MAF).Except EV-GRS, other three GRS caculations just simply consider the influence of SNP locus but ignore the effect of MAF that may have a very important part in the performance of the GRS method.

MAF is a frequency which is the second most common allele exsiting in the given population. It plays a surprising part in heritability since MAF variants which occurs only once, known as “singletons”, drive a huge amount of the selection^25^. MAF is very widely used in the population genetics research. It provides information that can differentiate the common and the rare variants in population^38^. Therefore, we think that MAF also plays important role in the construction of GRS. So we selected the EV-GRS to represent genetic risk factors and combined EV-GRS with traditional clinic risk factors to establish the ANN model.

And stoke extracted as the second important risk factor in our ANN according to Fig. 2. Stroke is the leading cause of death and disability in the whole world and is also an emergent public health problem^39^. A high level of plasma Hcy is proved to be an independent risk factor to stroke, and patients with HHcy will have a higher risk to develop stroke^40^. Stroke in HHcy patients is one of the major causes of morbidity and mortality. In addition, baseline Hcy was turned out to be the third important risk factor in our ANN model. The patients enrolled in our study all measured their plasma tHcy on the first day they participated in our research. Then to test the folic acid’s efficacy to reduce the Hcy level in plasma, the patients were supplied with 90 days’ oral folic acid (5 mg/day). In theory, the higher level of baseline Hcy the patients got, the more possibility to fail to reduce the Hcy level the patients would have. That may be the reason why baseline Hcy was turned out to be the third important risk factor in our ANN model.

Accordingly, we applied ANN to successfully establish an efficacy prediction model of folic acid’s therapy to HHcy. However, when comparing with the traditional multivariable LR model, ANN has several disadvantages^6^. First of all, ANN has a ‘black box’ nature; that is to say, ANN can’t clarify any insights into the structure of the function being approximated^41^. It is in contrast with the traditional LR model which can offer such information. Secondly, ANN has the risk of overtraining and the possibility of overfitting which may offer an overfitting prediction^42^. Finally, to clinical applications, ANN requires special statistical analysis software which may limit our model’s generalization and would be difficult to apply our model widely. However, Pergialiotis et al.^2^ clarified that these problems are able to be solved by using a larger number of participants (exclude the need for special statistical analysis software) as the small data set may not be applied to larger cohorts while the reverse is always very possible. Therefore, the establishment of the larger databases, for example, the database in a multicenter study, is very necessary for the establishment of a safer ANN model.

However, our study still had several limitations. First of all, our study was conducted in a single center. Secondly, the risk factors (age, diabetics, and the methylation level at some promoter regions) which have been previously reported to be associated with HHcy had not been enrolled in the establishment of our ANN model^35^. These factors were turned out to be not associated with our HHcy patients or were not tested in our research. In addition, the sample size of our research was relatively small which may limit the generalization of our ANN model to multiple populations. Therefore, further study is needed to be done to validate the efficacy of our ANN model in a bigger external cohort population.

In this study, we combined EV-GRS with ANN to predict the efficacy of oral folic acid treatment to HHcy. And the model exhibited good predictive performance. Therefore, our study indicates the application of ANN as a risk prediction model of folic acid therapy to HHcy patients in clinical practice. This model would be able to offer clinicians and pharmacists a new method to make decisions and individual therapeutic plans. Furthermore, several advanced ANN algorithms as a convolutional neural network, recursive neural network, recurrent neural network, and radial basis neural network, can also be employed for this purpose in further study. Thus, a more reliable prediction model would be constructed by performing the multicenter study and using more advanced ANN algorithms.

## Supplementary Information

Supplementary Table S1.
