# Supplementary material for: Combining genetic risk score with artificial neural network to predict the efficacy of folic acid therapy to hyperhomocysteinemia
Source: Sci Rep. 2021 Nov 2;11:21430. doi: 10.1038/s41598-021-00938-8 (PMC8563886; doi:10.1038/s41598-021-00938-8)
Supplement: Supplementary file 1 — Supplementary Table S1. [file 41598_2021_938_MOESM1_ESM.pdf]

Combining Genetic Risk Score with Artificial Neural Network to Predict the Efficacy of Folic Acid Therapy to Hyperhomocysteinemia

Running Title: Combining GRS with ANN to Predict the Efficacy to HHcy

Xiaorui Chen[1], Xiaowen Huang[1], Diao Jie[2], Caifang Zheng[1], Xiliang Wang[1], Bowen Zhang[1], Weihao Shao[1], Gaili Wang[1], and Weidong Zhang[1]*

[1]Department of Epidemiology, School of Public Health, Zhengzhou University, Zhengzhou, Henan, China.

[2]The University of Glasgow, G12 8QQ, Scotland, UK.

***Corresponding author**: Weidong Zhang, Department of Epidemiology, School of Public Health, Zhengzhou University, Zhengzhou, Henan, 450001, China;

Tel.: +86-0371-67781964; E-mail: imooni@163.com.

Supplemental Table 1 Information of candidate SNP loci associated with the efficacy of folic acid therapy to HHcy

Gene	SNP	Chr	Chr.Location	Risk/other allele	MAF	β	OR (95% CI)	P
MTHFR	rs1801133	1	11796321	T/C	0.3246	0.526	1.692 (1.298-2.205)	＜0.001*
MTHFR	rs1801131	1	11794419	A/C	0.2277	0.496	1.643 (1.118-2.413)	0.001*
MTHFR	rs2274976	1	11790870	A/G	0.05096	0.274	1.028(0.738-1.564)	0.986
MTHFD	rs2236225	14	64442127	G/A	0.35	0.427	1.583(1.277-1.993)	0.028*
MTHFD	rs1950902	14	64415662	C/T	0.191	0.301	1.042(0.839-1.347)	0.06
MTR	rs1805087	1	236885200	G/A	0.1933	0.236	1.266(0.814-1.971)	0.756
MTR	rs1050993	1	236899005	A/G	0.2645	0.196	1.193(0.648-1.432)	0.271
MTR	rs2275565	1	236885376	A/C	0.264	0.357	1.455(0.714-1.788)	0.399
MTR	rs12354209	1	236795952	G/A	0.3488	0.338	1.241(0.845-1.367)	0.154
MTR	rs1266164	1	236887651	C/T	0.3378	0.283	1.195(0.901-1.516)	0.719
MTR	rs2853523	1	236898898	A/C	0.3595	0.331	1.247(0.842-1.327)	0.645
MTRR	rs1801394	5	7870860	A/G	0.376	0.461	1.599(1.301-2.333)	＜0.001*
MTRR	rs1532268	5	7878066	G/A	0.2539	0.236	1.446(0.793-1.649)	0.081
MTRR	rs162036	5	7885846	A/G	0.2309	0.152	1.164(0.823-1.647)	0.081
MTRR	rs10380	5	7897078	C/T	0.115	0.315	1.284(0.744-1.639)	0.671
MTRR	rs12347	5	7897170	G/A	0.131	0.14	1.004(0.652-1.147)	0.792
MTRR	rs16879355	5	7892900	C/T	0.001	0.07	1.722(0.616-1.473)	0.824
MTRR	rs2287780	5	7889191	C/T	0.037	0.516	1.382(0.716-1.358)	0.074
CBS	rs2851391	21	43067294	C/T	0.399	0.274	1.315 (0.998-1.733)	0.065
CBS	rs706209	21	43065240	A/G	0.197	0.036	1.709 (1.252-2.332)	0.044*
CBS	rs234706	21	43065240	G/A	0.3304	0.412	1.244(0.910-1.503)	0.081
BHMT	rs3733890	5	79126136	A/G	0.3012	0.309	1.362 (1.022-1.815)	0.008*
BHMT	rs585800	5	79131385	A/T	0.1478	0.228	1.097(0.881-1.323）	0.069

MTHFR, methylenetetrahydrofolate reducaste; MTHFD, methylenetetrahydrofolate dehydrogenase; MTR, methionine synthase; MTRR, methionine synthase reductase; CBS, Cystathionine-β-synthase; BHMT, Betaine-homocysteine Methyltransferase; SNP, Single Nucleotide Polymorphism; Chr, Chromosomes; MAF, Minor Allele Frequency; OR, odds ratio.

*, significantly different.
